# A monoclinic polymorph of (nitrato-κ*O*)tetra­phenyl­anti­mony(V)

**DOI:** 10.1107/S1600536808040816

**Published:** 2008-12-06

**Authors:** Li Quan, Handong Yin, Daqi Wang

**Affiliations:** a College of Chemistry and Chemical Engineering, Liaocheng University, Shandong 252059, People’s Republic of China

## Abstract

The asymmetric unit of the title compound, [Sb(C_6_H_5_)_4_(NO_3_)], contains two crystallographically independent mol­ecules. Each Sb atom exhibits a slightly distorted trigonal-bipyramidal geometry, with the O atom in the apical site. The crystal structure is stabilized by inter­molecular C—H⋯O hydrogen bonds, forming a three-dimensional network.

## Related literature

For the structure of the triclinic polymorph, see: Sharutin *et al.* (2002 ▶). For the synthesis and structures of related triphenyl­anti­mony compounds, see: Yin *et al.* (2008 ▶); Chaudhari *et al.* (2007 ▶); Mahon *et al.* (1998 ▶); Liu *et al.* (2003 ▶).
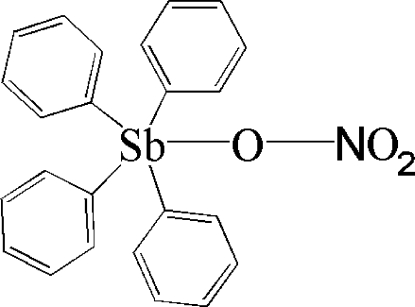


## Experimental

### 

#### Crystal data

[Sb(C_6_H_5_)_4_(NO_3_)]
*M* *_r_* = 492.17
Monoclinic, 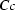

*a* = 16.7500 (19) Å
*b* = 12.0274 (12) Å
*c* = 22.561 (2) Å
β = 110.054 (2)°
*V* = 4269.5 (7) Å^3^
*Z* = 8
Mo *K*α radiation
μ = 1.32 mm^−1^
*T* = 298 (2) K
0.50 × 0.26 × 0.23 mm

#### Data collection

Bruker SMART diffractometer
Absorption correction: multi-scan (*SADABS*; Sheldrick, 1996 ▶) *T*_min_ = 0.548, *T*_max_ = 0.737
10559 measured reflections
5811 independent reflections
5258 reflections with *I* > 2σ(*I*)
*R*_int_ = 0.028

#### Refinement

*R*[*F* ^2^ > 2σ(*F* ^2^)] = 0.030
*wR*(*F* ^2^) = 0.056
*S* = 1.02
5811 reflections
523 parameters
3 restraints
H-atom parameters constrained
Δρ_max_ = 0.53 e Å^−3^
Δρ_min_ = −0.36 e Å^−3^
Absolute structure: Flack (1983 ▶), 2054 Friedel pairs
Flack parameter: −0.033 (18)

### 

Data collection: *SMART* (Siemens, 1996 ▶); cell refinement: *SAINT* (Siemens, 1996 ▶); data reduction: *SAINT*; program(s) used to solve structure: *SHELXS97* (Sheldrick, 2008 ▶); program(s) used to refine structure: *SHELXL97* (Sheldrick, 2008 ▶); molecular graphics: *ORTEP-3* (Farrugia, 1997 ▶) and *DIAMOND* (Brandenburg, 1998 ▶); software used to prepare material for publication: *SHELXTL* (Sheldrick, 2008 ▶).

## Supplementary Material

Crystal structure: contains datablocks I, global. DOI: 10.1107/S1600536808040816/rz2272sup1.cif

Structure factors: contains datablocks I. DOI: 10.1107/S1600536808040816/rz2272Isup2.hkl

Additional supplementary materials:  crystallographic information; 3D view; checkCIF report

## Figures and Tables

**Table 1:** Selected bond lengths (Å)

Sb1—C7	2.091 (5)
Sb1—C19	2.108 (5)
Sb1—C13	2.111 (5)
Sb1—C1	2.140 (6)
Sb1—O1	2.600 (4)
Sb2—C37	2.105 (5)
Sb2—C31	2.106 (5)
Sb2—C43	2.115 (5)
Sb2—C25	2.139 (6)
Sb2—O4	2.435 (4)

**Table 2:** Hydrogen-bond geometry (Å, °)

*D*—H⋯*A*	*D*—H	H⋯*A*	*D*⋯*A*	*D*—H⋯*A*
C27—H27⋯O3	0.93	2.48	3.366 (8)	160
C45—H45⋯O2^i^	0.93	2.55	3.429 (10)	157
C9—H9⋯O6^ii^	0.93	2.57	3.233 (8)	129
C40—H40⋯O3^iii^	0.93	2.55	3.449 (8)	163
C12—H12⋯O5^iv^	0.93	2.55	3.255 (7)	133
C33—H33⋯O3^v^	0.93	2.57	3.414 (8)	150

Symmetry codes: (i) 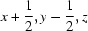; (ii) 
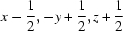
; (iii) 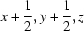; (iv) 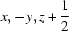; (v) 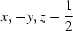.

**Fig. 1.**
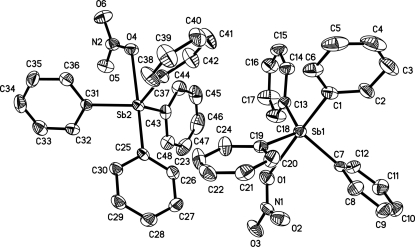
The molecular structure of the title compound, showing displacement ellipsoids drawn at the 30% probability level. H atoms are omitted for clarity.

**Fig. 2.**
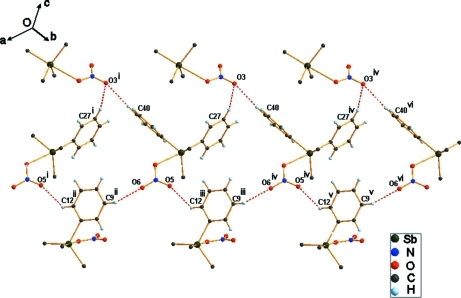
A partial packing diagram of the title compound showing the formation of chains linked by C—H···O interactions (dotted lines) [Symmetry codes: (i) = 1/2+x, 1/2+y, z; (ii) = 1/2+x, 1/2-y, -1/2+z; (iii) = x, -y, -1/2+z; (iv) = -1/2+x, -1/2+y, z; (v) -1/2+x, -1/2-y -1/2+z; (vi) -1+x, -1+y, z].

**Fig. 3.**
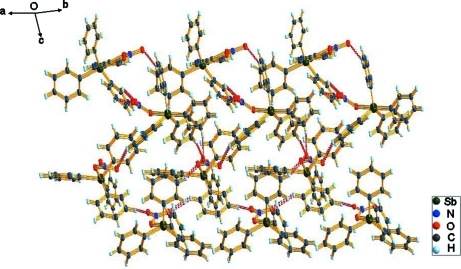
Packing diagram of the title compound showing the three-dimensional network. Intermolecular C—H···O hydrogen bonds are shown as dotted lines.

## supplementary crystallographic information

### Comment

Recently some organoantimony compounds have been reported by our group to possess potential biological activities (Yin *et al.*, 2008). In a continuation of this study, we had synthesized the title compound and report its crystal structure herein. The crystal structure of a first polymorph of the title compound has been previously reported by Sharutin *et al.* (2002) in the triclinic space group *P*1, with *a* = 10.002 (2), *b* = 12.332 (2), *c* = 18.758 (5) Å, *α* = 73.54 (2), *β* = 75.59 (2), *γ* = 81.78 (2)°.

As shown in Fig. 1, each antimony atom displays a slightly distorted trigonal bipyramidal geometry, with the equatorial positions occupied by the C atoms of three phenyl groups and the axial positions by one C atom of a phenyl group and one oxygen atom of the nitrate anion. The average Sb—C distance (2.114 Å; Table 1) corresponds well to that found in the triclinic polymorph, and is significantly shorter than the average distance found in [µ_2_-oxo)-bis((1-phenyl-3-methyl-4-benzoylpyrazolan-5-ato)-triphenyl- antimony(v)] (2.225 Å; Mahon *et al.*, 1998). The average Sb—O distance (2.518 Å) is comparable to that found in the triclinic polymorph (2.521 Å) and remarkably longer than the average distance found in [Me_3_Sb(O_2_C–C_5_H_4_N)_2_].H_2_O (2.145 Å; Chaudhari *et al.*, 2007). In the crystal structure, molecules are linked by C—H···O hydrogen bonds (Table 2) forming chains along the [1 1 0] direction (Fig. 2). The chains are further connected by additional C—H···O hydrogen bonds forming a three-dimensional network (Fig. 3).

### Experimental

Silver nitrate (0.339 g, 0.2 mmol) was added to a stirred solution of terraphenylantimonybromide (1.020 g, 0.2 mmol) in toluene (25 ml). After refluxing for 8 h, the resulting black mixture was filtered and the solvent gradually removed by evaporation under vacuum until a white solid was obtained. Colourless crystals suitable for X-ray analysis were obtained by slow evaporation of a petroleum ether/dichloromethane (1:1 v/v) solution.

### Refinement

All H atoms were positioned geometrically and refined as riding atoms, with C—H = 0.93 Å and U_iso_(H) = 1.2 U_eq_(C).

### Crystal data

**Table:**

[Sb(C_6_H_5_)_4_(NO_3_)]	*F*(000) = 1968
*M**_r_* = 492.17	*D*_x_ = 1.531 Mg m^−^^3^
Monoclinic, *C**c*	Mo *K*α radiation, λ = 0.71073 Å
Hall symbol: C -2yc	Cell parameters from 4801 reflections
*a* = 16.7500 (19) Å	θ = 2.5–25.5°
*b* = 12.0274 (12) Å	µ = 1.32 mm^−^^1^
*c* = 22.561 (2) Å	*T* = 298 K
β = 110.054 (2)°	Block, colourless
*V* = 4269.5 (7) Å^3^	0.50 × 0.26 × 0.23 mm
*Z* = 8	

### Data collection

**Table:**

Bruker SMART diffractometer	5811 independent reflections
Radiation source: fine-focus sealed tube	5258 reflections with *I* > 2σ(*I*)
graphite	*R*_int_ = 0.028
φ and ω scans	θ_max_ = 25.0°, θ_min_ = 1.9°
Absorption correction: multi-scan (*SADABS*; Sheldrick, 1996)	*h* = −11→19
*T*_min_ = 0.548, *T*_max_ = 0.737	*k* = −14→13
10559 measured reflections	*l* = −26→26

### Refinement

**Table:**

Refinement on *F*^2^	Secondary atom site location: difference Fourier map
Least-squares matrix: full	Hydrogen site location: inferred from neighbouring sites
*R*[*F*^2^ > 2σ(*F*^2^)] = 0.030	H-atom parameters constrained
*wR*(*F*^2^) = 0.056	*w* = 1/[σ^2^(*F*_o_^2^) + (0.018*P*)^2^] where *P* = (*F*_o_^2^ + 2*F*_c_^2^)/3
*S* = 1.02	(Δ/σ)_max_ = 0.001
5811 reflections	Δρ_max_ = 0.53 e Å^−^^3^
523 parameters	Δρ_min_ = −0.36 e Å^−^^3^
3 restraints	Absolute structure: Flack (1983), 2054 Friedel pairs
Primary atom site location: structure-invariant direct methods	Flack parameter: −0.033 (18)

### Special details

**Table:**

Geometry. All esds (except the esd in the dihedral angle between two l.s. planes) are estimated using the full covariance matrix. The cell esds are taken into account individually in the estimation of esds in distances, angles and torsion angles; correlations between esds in cell parameters are only used when they are defined by crystal symmetry. An approximate (isotropic) treatment of cell esds is used for estimating esds involving l.s. planes.
Refinement. Refinement of F^2^ against ALL reflections. The weighted R-factor wR and goodness of fit S are based on F^2^, conventional R-factors R are based on F, with F set to zero for negative F^2^. The threshold expression of F^2^ > 2sigma(F^2^) is used only for calculating R-factors(gt) etc. and is not relevant to the choice of reflections for refinement. R-factors based on F^2^ are statistically about twice as large as those based on F, and R- factors based on ALL data will be even larger.

### Fractional atomic coordinates and isotropic or equivalent isotropic displacement parameters (Å^2^)

**Table:**

	*x*	*y*	*z*	*U*_iso_*/*U*_eq_	
Sb1	0.28494 (2)	0.37970 (3)	0.346834 (16)	0.03919 (10)	
Sb2	0.261037 (18)	0.14794 (3)	0.063529 (14)	0.03867 (10)	
N1	0.0924 (3)	0.2489 (4)	0.2885 (2)	0.0527 (12)	
N2	0.4137 (3)	0.0435 (4)	0.0215 (2)	0.0521 (13)	
O1	0.1687 (3)	0.2327 (3)	0.2935 (2)	0.0587 (12)	
O2	0.0708 (3)	0.3394 (4)	0.3024 (2)	0.0726 (13)	
O3	0.0418 (3)	0.1706 (3)	0.26967 (19)	0.0743 (14)	
O4	0.4020 (3)	0.1219 (3)	0.05705 (18)	0.0508 (10)	
O5	0.3628 (3)	−0.0334 (3)	0.0056 (2)	0.0629 (11)	
O6	0.4767 (3)	0.0503 (4)	0.0058 (2)	0.0801 (15)	
C1	0.3748 (4)	0.5073 (4)	0.3908 (3)	0.0488 (15)	
C2	0.4043 (4)	0.5155 (5)	0.4552 (3)	0.0648 (19)	
H2	0.3876	0.4633	0.4790	0.078*	
C3	0.4584 (5)	0.5999 (7)	0.4850 (4)	0.092 (3)	
H3	0.4787	0.6047	0.5288	0.111*	
C4	0.4818 (5)	0.6762 (7)	0.4497 (6)	0.108 (4)	
H4	0.5158	0.7357	0.4698	0.130*	
C5	0.4570 (6)	0.6679 (6)	0.3862 (6)	0.096 (3)	
H5	0.4770	0.7180	0.3632	0.115*	
C6	0.4013 (4)	0.5836 (5)	0.3556 (3)	0.0682 (19)	
H6	0.3819	0.5786	0.3118	0.082*	
C7	0.2344 (3)	0.3543 (4)	0.4186 (2)	0.0374 (12)	
C8	0.1843 (4)	0.4354 (5)	0.4318 (3)	0.0566 (16)	
H8	0.1707	0.4997	0.4074	0.068*	
C9	0.1546 (4)	0.4210 (6)	0.4809 (3)	0.0676 (19)	
H9	0.1199	0.4746	0.4893	0.081*	
C10	0.1763 (5)	0.3269 (6)	0.5178 (3)	0.065 (2)	
H10	0.1563	0.3170	0.5511	0.078*	
C11	0.2272 (5)	0.2479 (5)	0.5053 (3)	0.0671 (19)	
H11	0.2432	0.1856	0.5311	0.081*	
C12	0.2550 (4)	0.2602 (5)	0.4549 (3)	0.0542 (16)	
H12	0.2876	0.2048	0.4455	0.065*	
C13	0.3632 (4)	0.2493 (4)	0.3360 (3)	0.0427 (14)	
C14	0.4459 (4)	0.2747 (5)	0.3434 (3)	0.0547 (16)	
H14	0.4644	0.3478	0.3515	0.066*	
C15	0.5023 (4)	0.1937 (5)	0.3390 (3)	0.0670 (18)	
H15	0.5576	0.2125	0.3426	0.080*	
C16	0.4757 (5)	0.0865 (6)	0.3295 (3)	0.074 (2)	
H16	0.5129	0.0313	0.3261	0.089*	
C17	0.3960 (5)	0.0597 (5)	0.3249 (4)	0.094 (3)	
H17	0.3783	−0.0140	0.3188	0.113*	
C18	0.3416 (4)	0.1387 (5)	0.3291 (4)	0.075 (2)	
H18	0.2874	0.1178	0.3272	0.090*	
C19	0.2101 (4)	0.4676 (4)	0.2660 (3)	0.0411 (14)	
C20	0.1778 (4)	0.5687 (5)	0.2743 (3)	0.0570 (17)	
H20	0.1923	0.5992	0.3144	0.068*	
C21	0.1238 (5)	0.6253 (5)	0.2229 (4)	0.063 (2)	
H21	0.1012	0.6934	0.2287	0.075*	
C22	0.1035 (4)	0.5821 (6)	0.1644 (3)	0.0652 (18)	
H22	0.0667	0.6200	0.1298	0.078*	
C23	0.1376 (5)	0.4822 (6)	0.1561 (3)	0.077 (2)	
H23	0.1241	0.4528	0.1157	0.093*	
C24	0.1914 (5)	0.4252 (5)	0.2067 (3)	0.0620 (18)	
H24	0.2149	0.3580	0.2006	0.074*	
C25	0.1390 (4)	0.1929 (4)	0.0666 (3)	0.0388 (13)	
C26	0.1174 (4)	0.1793 (4)	0.1207 (3)	0.0482 (14)	
H26	0.1564	0.1477	0.1566	0.058*	
C27	0.0388 (4)	0.2124 (5)	0.1214 (3)	0.0581 (17)	
H27	0.0246	0.2020	0.1574	0.070*	
C28	−0.0182 (4)	0.2607 (5)	0.0686 (3)	0.0634 (18)	
H28	−0.0710	0.2834	0.0693	0.076*	
C29	0.0012 (4)	0.2757 (5)	0.0155 (3)	0.0590 (17)	
H29	−0.0380	0.3084	−0.0200	0.071*	
C30	0.0799 (4)	0.2420 (4)	0.0145 (3)	0.0473 (14)	
H30	0.0932	0.2526	−0.0219	0.057*	
C31	0.2257 (3)	0.1317 (4)	−0.0351 (2)	0.0374 (12)	
C32	0.1621 (4)	0.0557 (4)	−0.0652 (3)	0.0491 (15)	
H32	0.1331	0.0186	−0.0426	0.059*	
C33	0.1425 (5)	0.0358 (5)	−0.1281 (3)	0.0638 (18)	
H33	0.1006	−0.0159	−0.1480	0.077*	
C34	0.1831 (5)	0.0900 (6)	−0.1621 (3)	0.075 (2)	
H34	0.1695	0.0747	−0.2049	0.090*	
C35	0.2448 (5)	0.1682 (6)	−0.1332 (3)	0.0696 (19)	
H35	0.2713	0.2072	−0.1569	0.084*	
C36	0.2670 (4)	0.1886 (5)	−0.0696 (3)	0.0538 (16)	
H36	0.3093	0.2400	−0.0500	0.065*	
C37	0.3313 (4)	0.2876 (4)	0.1089 (3)	0.0415 (14)	
C38	0.3054 (5)	0.3906 (5)	0.0836 (3)	0.081 (2)	
H38	0.2579	0.3977	0.0472	0.097*	
C39	0.3499 (6)	0.4832 (6)	0.1123 (4)	0.096 (3)	
H39	0.3347	0.5525	0.0934	0.115*	
C40	0.4163 (5)	0.4753 (5)	0.1679 (4)	0.076 (2)	
H40	0.4441	0.5389	0.1882	0.092*	
C41	0.4408 (6)	0.3744 (6)	0.1930 (3)	0.085 (3)	
H41	0.4868	0.3680	0.2303	0.102*	
C42	0.3982 (5)	0.2797 (5)	0.1635 (3)	0.076 (2)	
H42	0.4156	0.2103	0.1814	0.092*	
C43	0.2844 (4)	0.0027 (4)	0.1198 (2)	0.0464 (15)	
C44	0.3643 (5)	−0.0344 (5)	0.1516 (3)	0.0638 (19)	
H44	0.4115	0.0028	0.1488	0.077*	
C45	0.3738 (7)	−0.1310 (7)	0.1892 (4)	0.087 (3)	
H45	0.4280	−0.1565	0.2121	0.104*	
C46	0.3045 (8)	−0.1871 (7)	0.1923 (4)	0.099 (3)	
H46	0.3116	−0.2507	0.2170	0.118*	
C47	0.2247 (6)	−0.1499 (5)	0.1589 (4)	0.079 (2)	
H47	0.1776	−0.1886	0.1609	0.095*	
C48	0.2133 (5)	−0.0553 (4)	0.1223 (3)	0.0577 (17)	
H48	0.1589	−0.0303	0.0995	0.069*	

### Atomic displacement parameters (Å^2^)

**Table:**

	*U*^11^	*U*^22^	*U*^33^	*U*^12^	*U*^13^	*U*^23^
Sb1	0.0355 (2)	0.03932 (17)	0.0427 (2)	0.00308 (18)	0.01348 (18)	−0.00078 (17)
Sb2	0.0315 (2)	0.03843 (17)	0.0414 (2)	0.00229 (18)	0.00652 (17)	−0.00081 (17)
N1	0.037 (3)	0.065 (3)	0.045 (3)	−0.008 (3)	0.001 (3)	0.006 (3)
N2	0.040 (3)	0.054 (3)	0.061 (4)	0.010 (3)	0.016 (3)	0.007 (3)
O1	0.055 (3)	0.052 (2)	0.063 (3)	−0.002 (2)	0.011 (3)	−0.009 (2)
O2	0.049 (3)	0.061 (3)	0.102 (4)	0.004 (2)	0.019 (3)	−0.014 (2)
O3	0.066 (3)	0.070 (3)	0.066 (3)	−0.032 (3)	−0.005 (3)	0.006 (2)
O4	0.038 (2)	0.055 (2)	0.060 (3)	0.000 (2)	0.017 (2)	−0.012 (2)
O5	0.051 (3)	0.048 (2)	0.088 (3)	0.002 (2)	0.021 (3)	−0.012 (2)
O6	0.066 (3)	0.087 (3)	0.106 (4)	0.004 (3)	0.055 (3)	−0.003 (3)
C1	0.037 (4)	0.036 (3)	0.072 (5)	−0.002 (3)	0.017 (3)	−0.011 (3)
C2	0.047 (4)	0.071 (4)	0.067 (5)	−0.013 (4)	0.008 (4)	−0.026 (4)
C3	0.053 (5)	0.099 (6)	0.105 (7)	−0.011 (5)	0.002 (5)	−0.047 (5)
C4	0.059 (6)	0.083 (6)	0.183 (11)	−0.030 (5)	0.042 (7)	−0.063 (7)
C5	0.078 (6)	0.056 (4)	0.181 (10)	−0.020 (4)	0.077 (7)	−0.016 (5)
C6	0.057 (5)	0.058 (4)	0.099 (5)	−0.011 (3)	0.039 (4)	−0.004 (4)
C7	0.028 (3)	0.044 (3)	0.040 (3)	−0.004 (2)	0.011 (3)	0.002 (2)
C8	0.058 (4)	0.052 (3)	0.066 (4)	0.008 (3)	0.029 (4)	0.000 (3)
C9	0.066 (5)	0.070 (4)	0.078 (5)	−0.006 (4)	0.040 (4)	−0.022 (4)
C10	0.068 (5)	0.080 (5)	0.059 (4)	−0.023 (4)	0.036 (4)	−0.011 (4)
C11	0.075 (5)	0.065 (4)	0.068 (5)	−0.005 (4)	0.032 (4)	0.016 (3)
C12	0.049 (4)	0.060 (4)	0.061 (4)	−0.001 (3)	0.028 (4)	0.002 (3)
C13	0.041 (4)	0.042 (3)	0.048 (4)	0.007 (3)	0.019 (3)	−0.001 (2)
C14	0.043 (4)	0.054 (3)	0.070 (5)	0.001 (3)	0.022 (4)	−0.008 (3)
C15	0.043 (4)	0.077 (5)	0.088 (5)	0.004 (4)	0.031 (4)	−0.016 (4)
C16	0.058 (5)	0.071 (5)	0.094 (5)	0.020 (4)	0.027 (4)	−0.013 (4)
C17	0.077 (6)	0.044 (4)	0.172 (8)	0.006 (4)	0.058 (6)	−0.026 (4)
C18	0.053 (5)	0.051 (4)	0.124 (7)	−0.001 (3)	0.034 (5)	−0.018 (4)
C19	0.037 (4)	0.038 (3)	0.049 (4)	−0.001 (3)	0.014 (3)	0.004 (3)
C20	0.064 (5)	0.056 (4)	0.055 (4)	0.018 (3)	0.025 (4)	0.009 (3)
C21	0.056 (5)	0.059 (4)	0.078 (5)	0.022 (3)	0.029 (4)	0.019 (4)
C22	0.050 (4)	0.070 (4)	0.060 (5)	−0.008 (4)	−0.001 (4)	0.022 (4)
C23	0.102 (7)	0.075 (5)	0.039 (4)	−0.018 (5)	0.003 (4)	0.002 (3)
C24	0.085 (5)	0.051 (3)	0.045 (4)	0.001 (3)	0.015 (4)	−0.005 (3)
C25	0.036 (4)	0.034 (3)	0.040 (3)	0.003 (3)	0.005 (3)	−0.002 (2)
C26	0.047 (4)	0.050 (3)	0.043 (3)	0.003 (3)	0.009 (3)	−0.004 (3)
C27	0.057 (5)	0.068 (4)	0.057 (4)	0.000 (4)	0.030 (4)	−0.006 (3)
C28	0.049 (5)	0.071 (4)	0.073 (5)	0.010 (3)	0.025 (4)	−0.018 (4)
C29	0.040 (4)	0.072 (4)	0.055 (4)	0.019 (3)	0.003 (3)	−0.003 (3)
C30	0.040 (4)	0.053 (3)	0.046 (4)	0.008 (3)	0.010 (3)	−0.003 (3)
C31	0.031 (3)	0.040 (3)	0.038 (3)	0.006 (2)	0.009 (3)	0.002 (2)
C32	0.038 (4)	0.047 (3)	0.061 (4)	0.001 (3)	0.015 (3)	−0.005 (3)
C33	0.059 (5)	0.060 (4)	0.063 (5)	0.002 (3)	0.010 (4)	−0.020 (3)
C34	0.079 (6)	0.095 (5)	0.042 (4)	0.028 (5)	0.011 (4)	−0.011 (4)
C35	0.075 (5)	0.086 (5)	0.053 (4)	0.016 (4)	0.030 (4)	0.022 (4)
C36	0.048 (4)	0.055 (3)	0.054 (4)	−0.003 (3)	0.012 (3)	0.000 (3)
C37	0.041 (4)	0.036 (3)	0.042 (3)	0.001 (3)	0.007 (3)	−0.003 (2)
C38	0.075 (6)	0.048 (4)	0.087 (5)	−0.007 (4)	−0.015 (4)	−0.002 (3)
C39	0.114 (8)	0.054 (4)	0.094 (6)	−0.019 (5)	0.000 (6)	0.013 (4)
C40	0.090 (6)	0.056 (4)	0.073 (5)	−0.033 (4)	0.015 (5)	−0.017 (4)
C41	0.091 (7)	0.065 (5)	0.065 (5)	−0.019 (4)	−0.016 (4)	−0.012 (4)
C42	0.087 (6)	0.048 (4)	0.069 (5)	0.002 (4)	−0.006 (4)	0.001 (3)
C43	0.059 (4)	0.038 (3)	0.040 (4)	0.014 (3)	0.014 (3)	0.000 (3)
C44	0.062 (5)	0.062 (4)	0.066 (5)	0.021 (4)	0.020 (4)	0.009 (3)
C45	0.095 (8)	0.088 (6)	0.059 (5)	0.045 (5)	0.002 (5)	0.011 (4)
C46	0.164 (11)	0.067 (5)	0.073 (6)	0.029 (6)	0.051 (7)	0.017 (4)
C47	0.119 (8)	0.053 (4)	0.085 (6)	−0.002 (5)	0.060 (6)	0.009 (4)
C48	0.069 (5)	0.044 (3)	0.063 (4)	0.000 (3)	0.027 (4)	0.000 (3)

### Geometric parameters (Å, °)

**Table:**

Sb1—C7	2.091 (5)	C20—C21	1.381 (9)
Sb1—C19	2.108 (5)	C20—H20	0.9300
Sb1—C13	2.111 (5)	C21—C22	1.350 (9)
Sb1—C1	2.140 (6)	C21—H21	0.9300
Sb1—O1	2.600 (4)	C22—C23	1.370 (9)
Sb2—C37	2.105 (5)	C22—H22	0.9300
Sb2—C31	2.106 (5)	C23—C24	1.371 (9)
Sb2—C43	2.115 (5)	C23—H23	0.9300
Sb2—C25	2.139 (6)	C24—H24	0.9300
Sb2—O4	2.435 (4)	C25—C30	1.382 (7)
Sb2—O4	2.435 (4)	C25—C26	1.396 (7)
N1—O2	1.222 (6)	C26—C27	1.381 (8)
N1—O3	1.240 (6)	C26—H26	0.9300
N1—O3	1.240 (6)	C27—C28	1.374 (9)
N1—O1	1.259 (6)	C27—H27	0.9300
N2—O5	1.224 (6)	C28—C29	1.357 (8)
N2—O6	1.226 (6)	C28—H28	0.9300
N2—O4	1.296 (6)	C29—C30	1.387 (8)
N2—O4	1.296 (6)	C29—H29	0.9300
C1—C2	1.370 (8)	C30—H30	0.9300
C1—C6	1.381 (8)	C31—C36	1.386 (7)
C2—C3	1.373 (9)	C31—C32	1.392 (7)
C2—H2	0.9300	C32—C33	1.364 (8)
C3—C4	1.358 (12)	C32—H32	0.9300
C3—H3	0.9300	C33—C34	1.354 (9)
C4—C5	1.352 (12)	C33—H33	0.9300
C4—H4	0.9300	C34—C35	1.384 (10)
C5—C6	1.391 (10)	C34—H34	0.9300
C5—H5	0.9300	C35—C36	1.375 (8)
C6—H6	0.9300	C35—H35	0.9300
C7—C12	1.370 (7)	C36—H36	0.9300
C7—C8	1.385 (7)	C37—C42	1.355 (8)
C8—C9	1.372 (8)	C37—C38	1.371 (7)
C8—H8	0.9300	C38—C39	1.373 (8)
C9—C10	1.378 (9)	C38—H38	0.9300
C9—H9	0.9300	C39—C40	1.365 (10)
C10—C11	1.369 (9)	C39—H39	0.9300
C10—H10	0.9300	C40—C41	1.342 (9)
C11—C12	1.377 (8)	C40—H40	0.9300
C11—H11	0.9300	C41—C42	1.386 (8)
C12—H12	0.9300	C41—H41	0.9300
C13—C14	1.372 (8)	C42—H42	0.9300
C13—C18	1.373 (7)	C43—C44	1.358 (9)
C14—C15	1.384 (8)	C43—C48	1.398 (8)
C14—H14	0.9300	C44—C45	1.415 (10)
C15—C16	1.357 (9)	C44—H44	0.9300
C15—H15	0.9300	C45—C46	1.365 (12)
C16—C17	1.344 (10)	C45—H45	0.9300
C16—H16	0.9300	C46—C47	1.365 (12)
C17—C18	1.342 (8)	C46—H46	0.9300
C17—H17	0.9300	C47—C48	1.381 (8)
C18—H18	0.9300	C47—H47	0.9300
C19—C24	1.365 (7)	C48—H48	0.9300
C19—C20	1.369 (7)		
			
C7—Sb1—C19	117.7 (2)	C24—C19—C20	119.7 (6)
C7—Sb1—C13	114.42 (19)	C24—C19—Sb1	122.0 (4)
C19—Sb1—C13	118.7 (2)	C20—C19—Sb1	118.2 (4)
C7—Sb1—C1	98.8 (2)	C19—C20—C21	120.0 (6)
C19—Sb1—C1	99.0 (2)	C19—C20—H20	120.0
C13—Sb1—C1	102.7 (2)	C21—C20—H20	120.0
C7—Sb1—O1	79.68 (17)	C22—C21—C20	120.3 (6)
C19—Sb1—O1	79.16 (17)	C22—C21—H21	119.9
C13—Sb1—O1	80.63 (19)	C20—C21—H21	119.9
C1—Sb1—O1	176.65 (18)	C21—C22—C23	119.6 (6)
C37—Sb2—C31	119.7 (2)	C21—C22—H22	120.2
C37—Sb2—C43	114.2 (2)	C23—C22—H22	120.2
C31—Sb2—C43	118.98 (18)	C22—C23—C24	120.7 (6)
C37—Sb2—C25	99.4 (2)	C22—C23—H23	119.6
C31—Sb2—C25	97.6 (2)	C24—C23—H23	119.6
C43—Sb2—C25	99.9 (2)	C19—C24—C23	119.6 (6)
C37—Sb2—O4	74.92 (18)	C19—C24—H24	120.2
C31—Sb2—O4	81.46 (17)	C23—C24—H24	120.2
C43—Sb2—O4	86.89 (19)	C30—C25—C26	117.9 (5)
C25—Sb2—O4	172.60 (18)	C30—C25—Sb2	119.6 (4)
C37—Sb2—O4	74.92 (18)	C26—C25—Sb2	122.3 (4)
C31—Sb2—O4	81.46 (17)	C27—C26—C25	120.7 (6)
C43—Sb2—O4	86.89 (19)	C27—C26—H26	119.7
C25—Sb2—O4	172.60 (18)	C25—C26—H26	119.7
O4—Sb2—O4	0.0 (2)	C28—C27—C26	119.6 (6)
O2—N1—O3	122.5 (6)	C28—C27—H27	120.2
O2—N1—O3	122.5 (6)	C26—C27—H27	120.2
O2—N1—O1	119.8 (5)	C29—C28—C27	121.1 (6)
O3—N1—O1	117.8 (5)	C29—C28—H28	119.5
O3—N1—O1	117.8 (5)	C27—C28—H28	119.5
O5—N2—O6	123.2 (5)	C28—C29—C30	119.4 (6)
O5—N2—O4	119.5 (5)	C28—C29—H29	120.3
O6—N2—O4	117.3 (5)	C30—C29—H29	120.3
O5—N2—O4	119.5 (5)	C29—C30—C25	121.3 (6)
O6—N2—O4	117.3 (5)	C29—C30—H30	119.4
N1—O1—Sb1	120.8 (3)	C25—C30—H30	119.4
N2—O4—Sb2	119.1 (3)	C36—C31—C32	119.7 (5)
C2—C1—C6	119.5 (6)	C36—C31—Sb2	122.3 (4)
C2—C1—Sb1	118.9 (5)	C32—C31—Sb2	117.9 (4)
C6—C1—Sb1	121.6 (5)	C33—C32—C31	119.6 (6)
C1—C2—C3	120.7 (7)	C33—C32—H32	120.2
C1—C2—H2	119.7	C31—C32—H32	120.2
C3—C2—H2	119.7	C34—C33—C32	121.1 (6)
C4—C3—C2	119.1 (9)	C34—C33—H33	119.4
C4—C3—H3	120.4	C32—C33—H33	119.4
C2—C3—H3	120.4	C33—C34—C35	119.9 (6)
C5—C4—C3	121.7 (8)	C33—C34—H34	120.1
C5—C4—H4	119.1	C35—C34—H34	120.1
C3—C4—H4	119.1	C36—C35—C34	120.3 (6)
C4—C5—C6	119.3 (8)	C36—C35—H35	119.8
C4—C5—H5	120.3	C34—C35—H35	119.8
C6—C5—H5	120.3	C35—C36—C31	119.3 (6)
C1—C6—C5	119.5 (7)	C35—C36—H36	120.4
C1—C6—H6	120.2	C31—C36—H36	120.4
C5—C6—H6	120.2	C42—C37—C38	119.2 (6)
C12—C7—C8	120.0 (5)	C42—C37—Sb2	122.5 (4)
C12—C7—Sb1	119.8 (4)	C38—C37—Sb2	118.3 (5)
C8—C7—Sb1	120.0 (4)	C37—C38—C39	119.5 (7)
C9—C8—C7	119.9 (6)	C37—C38—H38	120.2
C9—C8—H8	120.0	C39—C38—H38	120.2
C7—C8—H8	120.0	C40—C39—C38	121.1 (6)
C8—C9—C10	119.8 (6)	C40—C39—H39	119.4
C8—C9—H9	120.1	C38—C39—H39	119.4
C10—C9—H9	120.1	C41—C40—C39	119.0 (6)
C11—C10—C9	120.0 (6)	C41—C40—H40	120.5
C11—C10—H10	120.0	C39—C40—H40	120.5
C9—C10—H10	120.0	C40—C41—C42	120.5 (7)
C10—C11—C12	120.4 (6)	C40—C41—H41	119.7
C10—C11—H11	119.8	C42—C41—H41	119.7
C12—C11—H11	119.8	C37—C42—C41	120.5 (6)
C7—C12—C11	119.7 (6)	C37—C42—H42	119.8
C7—C12—H12	120.2	C41—C42—H42	119.8
C11—C12—H12	120.2	C44—C43—C48	120.9 (6)
C14—C13—C18	116.4 (5)	C44—C43—Sb2	122.3 (5)
C14—C13—Sb1	117.5 (4)	C48—C43—Sb2	116.8 (4)
C18—C13—Sb1	125.7 (4)	C43—C44—C45	118.3 (8)
C13—C14—C15	121.4 (6)	C43—C44—H44	120.9
C13—C14—H14	119.3	C45—C44—H44	120.9
C15—C14—H14	119.3	C46—C45—C44	120.9 (8)
C16—C15—C14	119.0 (6)	C46—C45—H45	119.5
C16—C15—H15	120.5	C44—C45—H45	119.5
C14—C15—H15	120.5	C47—C46—C45	119.9 (7)
C17—C16—C15	120.2 (6)	C47—C46—H46	120.1
C17—C16—H16	119.9	C45—C46—H46	120.1
C15—C16—H16	119.9	C46—C47—C48	120.5 (8)
C16—C17—C18	120.4 (6)	C46—C47—H47	119.7
C16—C17—H17	119.8	C48—C47—H47	119.7
C18—C17—H17	119.8	C47—C48—C43	119.4 (7)
C13—C18—C17	122.4 (6)	C47—C48—H48	120.3
C13—C18—H18	118.8	C43—C48—H48	120.3
C17—C18—H18	118.8		
			
O2—N1—O1—Sb1	8.1 (7)	Sb1—C19—C20—C21	−175.5 (5)
O3—N1—O1—Sb1	−171.4 (3)	C19—C20—C21—C22	−1.0 (10)
O3—N1—O1—Sb1	−171.4 (3)	C20—C21—C22—C23	−0.6 (11)
C7—Sb1—O1—N1	55.1 (4)	C21—C22—C23—C24	0.6 (11)
C19—Sb1—O1—N1	−65.9 (4)	C20—C19—C24—C23	−2.7 (10)
C13—Sb1—O1—N1	172.2 (4)	Sb1—C19—C24—C23	175.5 (5)
O2—N1—O3—O3	0.00 (11)	C22—C23—C24—C19	1.0 (11)
O1—N1—O3—O3	0.00 (14)	C37—Sb2—C25—C30	97.3 (4)
O5—N2—O4—O4	0.00 (6)	C31—Sb2—C25—C30	−24.6 (4)
O6—N2—O4—O4	0.00 (17)	C43—Sb2—C25—C30	−145.9 (4)
O5—N2—O4—Sb2	−19.6 (6)	C37—Sb2—C25—C26	−79.6 (4)
O6—N2—O4—Sb2	161.1 (4)	C31—Sb2—C25—C26	158.5 (4)
O4—N2—O4—Sb2	0(26)	C43—Sb2—C25—C26	37.2 (5)
C37—Sb2—O4—O4	0.000 (9)	C30—C25—C26—C27	1.1 (8)
C31—Sb2—O4—O4	0.00 (4)	Sb2—C25—C26—C27	178.1 (4)
C43—Sb2—O4—O4	0.00 (3)	C25—C26—C27—C28	−1.0 (9)
C37—Sb2—O4—N2	−169.9 (4)	C26—C27—C28—C29	0.5 (9)
C31—Sb2—O4—N2	−45.8 (4)	C27—C28—C29—C30	−0.1 (9)
C43—Sb2—O4—N2	74.1 (4)	C28—C29—C30—C25	0.2 (9)
O4—Sb2—O4—N2	0(2)	C26—C25—C30—C29	−0.7 (8)
C7—Sb1—C1—C2	23.8 (5)	Sb2—C25—C30—C29	−177.8 (4)
C19—Sb1—C1—C2	143.8 (5)	C37—Sb2—C31—C36	20.3 (5)
C13—Sb1—C1—C2	−93.9 (5)	C43—Sb2—C31—C36	−128.5 (4)
C7—Sb1—C1—C6	−153.9 (5)	C25—Sb2—C31—C36	125.7 (4)
C19—Sb1—C1—C6	−33.9 (5)	O4—Sb2—C31—C36	−46.9 (4)
C13—Sb1—C1—C6	88.5 (5)	O4—Sb2—C31—C36	−46.9 (4)
C6—C1—C2—C3	1.3 (10)	C37—Sb2—C31—C32	−163.8 (4)
Sb1—C1—C2—C3	−176.4 (5)	C43—Sb2—C31—C32	47.5 (5)
C1—C2—C3—C4	0.6 (11)	C25—Sb2—C31—C32	−58.3 (4)
C2—C3—C4—C5	−3.6 (13)	O4—Sb2—C31—C32	129.1 (4)
C3—C4—C5—C6	4.6 (13)	O4—Sb2—C31—C32	129.1 (4)
C2—C1—C6—C5	−0.4 (9)	C36—C31—C32—C33	1.7 (8)
Sb1—C1—C6—C5	177.3 (5)	Sb2—C31—C32—C33	−174.4 (4)
C4—C5—C6—C1	−2.5 (11)	C31—C32—C33—C34	−1.0 (9)
C19—Sb1—C7—C12	148.2 (4)	C32—C33—C34—C35	−0.9 (10)
C13—Sb1—C7—C12	1.7 (5)	C33—C34—C35—C36	2.1 (10)
C1—Sb1—C7—C12	−106.7 (5)	C34—C35—C36—C31	−1.4 (10)
O1—Sb1—C7—C12	76.4 (4)	C32—C31—C36—C35	−0.5 (8)
C19—Sb1—C7—C8	−35.6 (5)	Sb2—C31—C36—C35	175.4 (4)
C13—Sb1—C7—C8	177.9 (4)	C31—Sb2—C37—C42	−136.7 (6)
C1—Sb1—C7—C8	69.5 (5)	C43—Sb2—C37—C42	13.4 (6)
O1—Sb1—C7—C8	−107.4 (5)	C25—Sb2—C37—C42	118.9 (6)
C12—C7—C8—C9	−0.6 (9)	O4—Sb2—C37—C42	−66.0 (6)
Sb1—C7—C8—C9	−176.8 (5)	O4—Sb2—C37—C42	−66.0 (6)
C7—C8—C9—C10	1.4 (10)	C31—Sb2—C37—C38	46.6 (6)
C8—C9—C10—C11	−0.1 (10)	C43—Sb2—C37—C38	−163.2 (5)
C9—C10—C11—C12	−2.0 (11)	C25—Sb2—C37—C38	−57.8 (5)
C8—C7—C12—C11	−1.5 (9)	O4—Sb2—C37—C38	117.3 (5)
Sb1—C7—C12—C11	174.7 (5)	O4—Sb2—C37—C38	117.3 (5)
C10—C11—C12—C7	2.8 (10)	C42—C37—C38—C39	3.1 (12)
C7—Sb1—C13—C14	−120.3 (4)	Sb2—C37—C38—C39	179.9 (6)
C19—Sb1—C13—C14	93.5 (5)	C37—C38—C39—C40	−4.4 (13)
C1—Sb1—C13—C14	−14.3 (5)	C38—C39—C40—C41	3.6 (14)
O1—Sb1—C13—C14	165.6 (5)	C39—C40—C41—C42	−1.5 (14)
C7—Sb1—C13—C18	52.0 (6)	C38—C37—C42—C41	−1.1 (12)
C19—Sb1—C13—C18	−94.2 (6)	Sb2—C37—C42—C41	−177.7 (6)
C1—Sb1—C13—C18	158.0 (6)	C40—C41—C42—C37	0.2 (13)
O1—Sb1—C13—C18	−22.1 (6)	C37—Sb2—C43—C44	−49.6 (5)
C18—C13—C14—C15	5.0 (10)	C31—Sb2—C43—C44	100.8 (5)
Sb1—C13—C14—C15	178.0 (5)	C25—Sb2—C43—C44	−154.8 (5)
C13—C14—C15—C16	−2.4 (10)	O4—Sb2—C43—C44	22.3 (5)
C14—C15—C16—C17	−0.6 (11)	O4—Sb2—C43—C44	22.3 (5)
C15—C16—C17—C18	0.7 (13)	C37—Sb2—C43—C48	131.2 (4)
C14—C13—C18—C17	−4.9 (11)	C31—Sb2—C43—C48	−78.4 (5)
Sb1—C13—C18—C17	−177.3 (6)	C25—Sb2—C43—C48	26.0 (4)
C16—C17—C18—C13	2.2 (13)	O4—Sb2—C43—C48	−156.9 (4)
C7—Sb1—C19—C24	−126.0 (5)	O4—Sb2—C43—C48	−156.9 (4)
C13—Sb1—C19—C24	19.1 (6)	C48—C43—C44—C45	−2.2 (9)
C1—Sb1—C19—C24	129.0 (5)	Sb2—C43—C44—C45	178.6 (5)
O1—Sb1—C19—C24	−53.8 (5)	C43—C44—C45—C46	1.6 (11)
C7—Sb1—C19—C20	52.2 (5)	C44—C45—C46—C47	−0.2 (12)
C13—Sb1—C19—C20	−162.8 (5)	C45—C46—C47—C48	−0.4 (12)
C1—Sb1—C19—C20	−52.8 (5)	C46—C47—C48—C43	−0.3 (10)
O1—Sb1—C19—C20	124.3 (5)	C44—C43—C48—C47	1.6 (9)
C24—C19—C20—C21	2.7 (9)	Sb2—C43—C48—C47	−179.1 (4)

### Hydrogen-bond geometry (Å, °)

**Table:**

*D*—H···*A*	*D*—H	H···*A*	*D*···*A*	*D*—H···*A*
C27—H27···O3	0.93	2.48	3.366 (8)	160
C45—H45···O2^i^	0.93	2.55	3.429 (10)	157
C9—H9···O6^ii^	0.93	2.57	3.233 (8)	129
C40—H40···O3^iii^	0.93	2.55	3.449 (8)	163
C12—H12···O5^iv^	0.93	2.55	3.255 (7)	133
C33—H33···O3^v^	0.93	2.57	3.414 (8)	150

Symmetry codes:  (i) *x*+1/2, *y*−1/2, *z*; (ii) *x*−1/2, −*y*+1/2, *z*+1/2; (iii) *x*+1/2, *y*+1/2, *z*; (iv) *x*, −*y*, *z*+1/2; (v) *x*, −*y*, *z*−1/2.

## References

[bb1] Brandenburg, K. (1998). *DIAMOND* Crystal Impact GbR, Bonn, Germany.

[bb2] Chaudhari, K. R., Jain, V. K., Sagoria, V. S. & Tiekink, E. R. T. (2007). *J. Organomet. Chem.* **692**, 4928–4932.

[bb3] Farrugia, L. J. (1997). *J. Appl. Cryst.* **30**, 565.

[bb4] Flack, H. D. (1983). *Acta Cryst.* A**39**, 876–881.

[bb5] Liu, R. C., Ma, Y. Q., Li, J. S., Cui, J. R. & Wang, R. Q. (2003). *Appl. Organomet. Chem.* **17**, 662–668.

[bb6] Mahon, M. F., Molloy, K. C., Omotowa, B. A. & Mesubi, M. A. (1998). *J. Organomet. Chem.* **560**, 95–101.

[bb7] Sharutin, V. V., Sharutina, O. K., Panova, L. P., Platonova, T. P., Pakusina, A. P., Krivolapov, D. B., Gubaidullin, A. T. & Litvinov, I. A. (2002). *Russ. J. Gen. Chem.* **72**, 40–43.

[bb8] Sheldrick, G. M. (1996). *SADABS* University of Göttingen, Germany.

[bb9] Sheldrick, G. M. (2008). *Acta Ctyst.* A**63**, 112–122.

[bb10] Siemens (1996). *SMART* and *SAINT* Siemens Analytical X-ray Instruments Inc., Madison, Wisconsin, USA.

[bb11] Yin, H. D., Quan, L. & Li, L. W. (2008). *Inorg. Chem. Commun.* **11**, 1122–1125.

